# Change Within Stability: A Mixed‐Methods Study of Identity Development in Established Adulthood

**DOI:** 10.1111/jopy.70046

**Published:** 2026-01-04

**Authors:** Hanna Larsson, Johanna Carlsson, Py Liv Eriksson, Moin Syed, Maria Wängqvist, Ann Frisén

**Affiliations:** ^1^ Department of Psychology University of Gothenburg Gothenburg Sweden; ^2^ Department of Psychology University of Minnesota Minneapolis Minnesota USA

**Keywords:** emerging adulthood, established adulthood, identity commitments, identity development, identity processes, identity status, longitudinal patterns

## Abstract

**Introduction:**

Longitudinal studies examining identity development in adulthood are scarce, and little is known about the processes through which a stable identity is maintained or revised. This study addresses these gaps by examining (a) longitudinal patterns of identity status development from emerging through established adulthood and (b) processes of continued identity development among individuals who are stable in committed identity statuses.

**Methods and Results:**

Quantitative analyses of identity status development across ages 25, 29, 33, and 39 (*N* = 105) revealed group‐level changes, with more individuals in identity achievement and fewer in moratorium and identity diffusion at age 39 than at younger ages. However, the largest group of participants (35%, *n* = 37) were assigned the same committed identity status at all four time points. Longitudinal qualitative analysis of this subset from ages 33 and 39 resulted in a model with three processes of continued identity development: *anchoring commitments in views o* *f oneself*, *story integration*, and *connecting oneself to older and younger generations*.

**Conclusion:**

The findings show that there is identity status change toward maturity from emerging through established adulthood, and that processes of continued development take place for individuals considered stable in their identity.

Identity is a central aspect of personality (McAdams and Pals 2006; Syed 2017), which continues to develop throughout the lifespan (Erikson 1968). Solidifying a sense of identity has been suggested as a key developmental aspect of established adulthood (around ages 30–45; Reifman and Niehuis 2022), a phase of life defined by increased stability, responsibility, and demands in the areas of work and family (Mehta et al. 2020). Previous research indicates that the proportion of individuals who have a stable sense of who they are and what they value in important life areas (i.e., have made identity‐defining commitments) increases during established adulthood (Eriksson et al. 2020; Fadjukoff et al. 2016; Kroger et al. 2010). However, longitudinal studies examining identity development across adulthood are scarce, and little is known about the processes through which a stable identity is maintained or re‐established when challenged (Mitchell et al. 2021). In the present study, we will address these gaps by examining (a) longitudinal patterns of identity status development from emerging through established adulthood and (b) processes of continued identity development among individuals who are stable in committed identity statuses.

## 1 Established Adulthood

The concept of established adulthood (around ages 30–45) describes a time of life that in the US and countries with similar demographic trends involves transitioning into social roles and taking on responsibilities traditionally associated with adulthood, such as settling into a career, establishing a long‐term romantic relationship, or becoming a parent (Mehta and Arnett 2023). In contrast to emerging adulthood (around ages 19–29; Arnett 2014), established adulthood is defined by greater stability and responsibility in both work and family life (Mehta et al. 2020). In Sweden, where the current study is situated, it is indeed during established adulthood that most people establish themselves on the job market and form families (Ekonomifakta 2018; Statistics Sweden 2023a, 2023b). Established adulthood can thus be defined as a time of role immersion and of juggling demands in both professional and family life, known as the *care‐and‐career crunch* (Arnett 2012; Mehta et al. 2020).

Many of the developmental tasks and central features of established adulthood can pose challenges to established adults' sense of identity. Taking on roles such as that of a spouse or parent may require individuals to explore new aspects of their identity, adjust their previous views of themselves, and integrate old and new identity elements into a meaningful whole (Laney et al. 2015; Pals 1999). Feeling discontent with or trapped in one's roles and commitments can lead to renewed identity exploration and life course corrections (Mehta and LaRiviere 2022; Robinson 2015). At the same time, some life changes may feel less attainable than earlier, for example career switches that demand extensive higher education (Reifman and Niehuis 2022), and responsibilities toward others can limit possibilities for self‐focus and exploration (Arnett 2014). Diverging from sociocultural norms regarding expected life course events, for example by remaining child free, can also challenge individuals' identities during this phase of life (e.g., McLean et al. 2018; Rubin and Berntsen 2003). Developing and maintaining a sense of identity thus continues to be important during established adulthood in many different ways, yet empirical studies of identity development extending beyond the emerging adult years are scarce (Fadjukoff and Kroger 2016).

## 2 Identity Status Development in Established Adulthood

One of the dominant approaches to studying identity development is Marcia's (1966) identity status model (McLean and Syed 2015). This model, based on Erikson's (1963, 1968) theory of identity, focuses on the *exploration* of different identity options and the establishment of identity‐defining *commitments* in important areas of life as two core processes for identity formation (Marcia 1966). Based on their degree of exploration and commitment, individuals can be categorized into four identity statuses: identity diffusion, foreclosure, moratorium, and identity achievement (Marcia 1966; Marcia et al. 1993). The identity diffusion and moratorium statuses are both defined by a lack of identity commitments but differ in that individuals in moratorium are in an active state of exploring identity alternatives in order to form commitments whereas individuals in identity diffusion are not (Kroger and Marcia 2011; Marcia 1966; Marcia et al. 1993). The foreclosed and identity achievement statuses are both characterized by the presence of clearly defined commitments but differ in the process through which their commitments were established; identity achieved individuals have explored different identity alternatives in order to arrive at their commitments, whereas individuals in foreclosure may instead have taken over commitments from their parents or other authority figures in their social context without much consideration of alternatives (Kroger and Marcia 2011; Marcia 1966; Marcia et al. 1993). From an identity status perspective, the optimal development from adolescence to adulthood would theoretically include exploration leading to commitments, resulting in a progressive movement toward identity achievement (Waterman 1999). However, in contrast to theoretical assumptions, studies have shown that the foreclosed identity status is common beyond adolescence (Eriksson et al. 2020; Fadjukoff et al. 2016; Kroger et al. 2010), and that this status is generally associated with high levels of well‐being and psychosocial functioning (for a summary, see Meeus 2011, 2018). Therefore, it has been suggested that it is the establishment of identity commitments that is the most central to an individual's development rather than how these commitments came to be (Meeus 2011, 2018).

Reviews of previous cross‐sectional and (a few) longitudinal studies conclude that progressive or stable patterns of development are more common than regressive ones across adolescence and adulthood, with the uncommitted statuses (i.e., identity diffusion and moratorium) tending to decrease over time while the committed statuses (i.e., foreclosure and achievement) tend to be stable or increase (Branje et al. 2021; Fadjukoff et al. 2016; Kroger et al. 2010; Meeus 2011). Moreover, individual stability in identity status seems to be more common in adulthood than in adolescence, which highlights an important difference between these age groups (Meeus 2011). In line with these findings, previous results from the same longitudinal project as the current study showed that remaining in identity achievement or foreclosure across ages 25, 29, and 33 was the most common developmental pattern (Eriksson et al. 2020). The relative stability in committed identity statuses observed across adulthood raises questions about what identity development looks like for adults after they have made commitments. The identity status model was originally created for investigating identity development in adolescents, with exploration and commitment conceptualized as processes for forming an identity rather than for continuing to develop this identity as an adult (Kroger 2015; Mitchell et al. 2021). Newer extensions of the identity status model have distinguished between processes involved in forming and maintaining identity (Crocetti et al. 2008; Luyckx et al. 2005). However, these models were not developed with identity during adulthood in mind, and empirical studies using them rely heavily on survey measures that are not sensitive to the specific developmental challenges of established adulthood. Gaining a contextualized understanding of how adults continue to develop their identities after having established identity commitments therefore calls for complementary perspectives and methods that go beyond the identity status paradigm.

## 3 Maintaining and Revising a Stable Adult Identity

Multiple perspectives on identity development converge on the idea that adults need to continuously apprehend, interpret, and integrate new identity‐relevant experiences in light of their already established sense of self in order to maintain a sense of wholeness and continuity (e.g., Erikson 1968; Marcia 2002; McAdams 2013; Mitchell et al. 2021). In their identity integration framework, Mitchell et al. (2021) conceptualized such processes of continued development in terms of two broad categories: processes involved in maintaining an integrated sense of identity in adulthood, and processes that re‐establish integration when it is challenged by normative or unexpected life events. According to this framework, identity maintenance processes could include deepening commitments to important aspects of one's identity and gradually adjusting one's identity to accommodate minor changes. Re‐establishment processes may include taking action to minimize the identity impact of challenging events and adopting new identity aspects that are better suited to the new situation. Moreover, Mitchell and colleagues suggest that both maintenance and re‐establishment can be achieved through narrative identity processes, which involve reflecting upon and integrating past and present experiences with the imagined future to create a coherent and meaningful life story (Habermas 2011; McAdams 2001). The identity integration framework is based on an extensive review of developmental, lifespan, and personality literature, and provides a useful conceptual map for exploring identity development in adulthood. However, empirical work is needed in order to understand people's lived experience of maintaining and re‐establishing their identity after having made identity commitments, how such processes unfold over time, and how they might differ between individuals.

A few previous qualitative studies have examined processes of continued identity development specifically among adults in committed identity statuses. Findings from the longitudinal project that the current study is part of highlighted how individuals continued to deepen or weaken their identity from age 25 to 29 (Carlsson et al. 2015) and 29 to 33 (Eriksson et al. 2020) in relation to three broad dimensions of development: their commitments, their identity narratives, and their approach to their social context. Between ages 25 and 29, prominent themes revolved around navigating individual development in relation to the external world, including how individuals adjusted their commitments to changing life conditions and developed their personal life direction in relation to social norms (Carlsson et al. 2015). Between ages 29 and 33, key processes instead included development in how individuals adjusted their commitments in response to internal needs and in how they included times, places, and people beyond their immediate personal context as important to their identity (Eriksson et al. 2020). Kroger (2002) examined processes of continued development among older adults and described, for example, how individuals maintained their identity by finding ways to retain personal interests despite diminished physical capacities and how they revised their identity by reintegrating important identity elements of the past. Taken together, these studies highlight that there might be age‐related variations in the processes by which individuals continue to develop their identities and in the developmental themes that are central to them across different phases of adulthood. In the current study, we will build on the work of Carlsson et al. (2015) and Eriksson et al. (2020) by examining processes of continued identity development for individuals who are stable in committed identity statuses specifically in relation to established adulthood, to better understand identity development in this phase of life.

## 4 The Present Study

The aim of this study was to investigate processes of identity development in established adulthood. We addressed this aim using an explanatory mixed‐methods design (Creswell 2021). The specific research questions examined were:

- What patterns of identity status change and stability can be seen across ages 25, 29, 33, and 39? (Section 2).
- What processes of continued identity development from age 33 to 39 can be identified among individuals who are stable in committed identity statuses (foreclosure or identity achievement) across ages 25, 29, 33, and 39? (Section 3).

## 1 General Method

### 1.1 Participants

This study is part of the Gothenburg Longitudinal study of Development (GoLD). GoLD started in 1982 with a community sample of 144 1‐ to 2‐year‐olds (see e.g., Broberg et al. 1990). Participants were recruited from waiting lists for public childcare services in different areas of Gothenburg, the second largest city in Sweden, and approximately 75% of the contacted families agreed to participate. These families had a variety of backgrounds, and the sample was largely representative of families living in Gothenburg at the time in terms of parental age and socioeconomic status (Lamb et al. 1988).

This study draws from Waves 8–11 of GoLD and includes all participants who completed the Identity Status Interview at all four time points (*N* = 105, 56 women and 49 men). The age of the included participants ranged from 24 to 26 years (M = 24.88, SD = 0.65) at Wave 8, 28 to 30 years (M = 29.24, SD = 0.60) at Wave 9, 32–34 years (M = 33.28, SD = 0.53) at Wave 10, and 38–40 years (M = 38.60, SD = 0.60) at Wave 11. All participants had grown up in Sweden and 88 (83.8%) of them also had parents who had been born in Sweden, whereas seven (6.7%) had at least one parent born in another Scandinavian country and 10 (9.6%) had at least one parent born outside Scandinavia. Changes in occupational status and family life could be observed across Waves 8–11 for participants included in this study (see Table 1; full results for McNemar analyses are available at OSF). These changes followed expected demographic developments; for example, finishing postsecondary education and entering qualified work positions was more common after age 25, and transitioning into parenthood became increasingly more common with age. Participants who had not completed the Identity Status Interview in all of the four most recent waves of data collection (*n* = 39), and thus were excluded from the sample, were not statistically different from included participants in terms of gender or parental socioeconomic status (Hollingshead 1975) at the start of the study (full analyses and additional information about participants who dropped out during the three most recent waves of the study is provided in Material S1 at OSF).

**TABLE 1:** Participants' occupational status and family life at ages 25, 29, 33, and 39—descriptive data, Cochran's *Q* test, and McNemar test of significance of change.

Measure	Age 25, *n* (%)	Age 29, *n* (%)	Age 33, *n* (%)	Age 39, *n* (%)	Cochran's *Q* ^a^	McNemar, *ꭓ* ^2^					
						25–29	29–33	33–39	25–33	25–39	29–39
Occupational status											
Student	45 (42.9)	7 (6.7)	5 (4.8)	3 (2.9)	90.6***	29.76***	—	—	36.21***	36.54***	—
Working after postseconday education	20 (19.0)	66 (62.9)	74 (70.5)	81 (77.1)	121.21***	42.19***	—	—	50.16***	55.39***	*p* = 0.003^c^
Working without postsecondary education	36 (34.3)	25 (23.8)	22 (21.0)	17 (16.2)	19.73***	—	—	—	—	*p* < 0.001^c^	—
Other^b^	4 (3.8)	7 (6.7)	4 (3.8)	4 (3.8)	1.53	—	—	—	—	—	—
Family life											
In a romantic relationship	69 (65.7)	84 (80.0)	90 (85.7)	93 (88.6)	26.65***	*p* = 0.004^c^	—	—	11.43***	13.92***	—
Cohabiting with a partner	53 (50.5)	70 (66.7)	83 (79.0)	85 (81.0)	39.76***	7.31**	—	—	21.03***	20.02***	—
Have or expect children	8 (7.6)	39 (37.1)	71 (67.6)	86 (81.9)	163.22***	29.03***	30.03***	*p* < 0.001^c^	61.02***	76.01***	45.02***

*Note:* *N* = 105 (56 women and 49 men).

^a^ Cochran's *Q* test for related proportions between three or more matched sets (Siegel and Castellan 1988), with post hoc McNemar test for significance of changes.

^b^ For example, unemployed or on long‐term sick leave.

^c^ Exact sig. (two‐tailed), binomial distribution used.

** *p* < 0.01.

*** *p* < 0.001.

### 1.2 Procedure

Before each wave of data collection, participants were given information about the study by letter and were thereafter contacted by telephone or e‐mail and asked if they were willing to participate. For detailed procedural information about each wave of data collection (e.g., number of participants, interviewer, interview location, etc.), see Material S2 at OSF.

### 1.3 Measures

#### 1.3.1 Background Interview

A structured background interview, including questions regarding the participants' occupational status and family situation, was performed at each wave of data collection.

#### 1.3.2 The Identity Status Interview

The Identity Status Interview (Marcia 1966; Marcia et al. 1993) was carried out at each wave of data collection. The interview has a semi‐structured format, and includes questions and probes aimed at uncovering the processes of exploration and commitment in various identity domains. Based on the degree of exploration and commitment expressed by the participants, they were assigned to either identity achievement, foreclosure, moratorium, or diffusion. For the present study, we used a version of the Identity Status Interview that had been translated into Swedish and adapted to Swedish conditions (Frisén and Wängqvist 2011). The domains examined were occupation, romantic relationships, parenthood, and work‐family priorities. Coding of identity status followed the procedure outlined by Marcia et al. (1993). This procedure involved first determining the participant's identity status in each domain, followed by an assessment of their global identity status. When there was incongruence across domains, the final determination of global identity status was based on a combination of the five guidelines described by Marcia et al. (1993). All interviewers had undergone training in conducting and coding the Identity Status Interview. In all waves, identity status assessments were performed by the interviewer who had met with the participant, but any uncertainties regarding the coding were discussed with the other interviewers until consensus was reached. To ensure reliability in the coding, a random sample of interviews (*n* = 20) was also re‐coded by an additional research team member at all four waves of data collection. The exact agreements for global identity status assessment were: at age 25, 85.0% with a kappa of 0.77 (Frisén and Wängqvist 2011); at age 29, 85.0% with a kappa of 0.72 (Carlsson et al. 2015); at age 33, 95.0% with a kappa of 0.89 (Gyberg and Frisén 2017); and at age 39, 95.0% with a kappa of 0.89.

Beyond providing information about identity status, the Identity Status Interview also elicits developmental narratives about how, when, and why an individual came to explore and/or commit to different identity alternatives (Marcia et al. 1993). This rich and contextualized information is not evident in the categorical assessment of identity status but can be analyzed using qualitative methods. In the qualitative part of this study, we utilized the Identity Status Interview to this end by examining changes in the narrative content of interviews from ages 33 and 39 for a subset of participants (see Section 3).

### 1.4 Analysis

An explanatory mixed‐methods design (Creswell 2021) was applied in the present study. The design was sequential, in which results from the quantitative analyses informed the sampling frame for the qualitative analysis. The quantitative part of the study is presented below, followed by the qualitative. The reporting on the research design and data analysis was guided by the JARS guidelines (Levitt et al. 2018).

## 2 Quantitative Study

The research question examined in the quantitative part of the study was: What patterns of identity status change and stability can be seen across ages 25, 29, 33, and 39?

### 2.1 Data Analysis

First, a variable‐centered approach was employed to examine group‐level stability and change in identity status across waves using Cochran's *Q* test followed by a post hoc McNemar test for significance of change (Siegel and Castellan 1988). Second, a person‐centered approach was employed to investigate typical and atypical patterns of individual stability and change in identity status between adjacent waves (i.e., from age 25 to 29, 29 to 33, and 33 to 39), using the cross‐tabulation procedure EXACON (Bergman et al. 2002). EXACON, built on the Fisher four‐field hypergeometric distribution test, identifies which individual patterns of change and stability occur more or less frequently than expected by chance. Cross‐tabulation chi‐square analysis was used to examine gender differences in identity status at each wave and in participants with typical patterns of development across all waves. A restricted alpha level of *p* < 0.01 was chosen for all analyses in order to adjust for the number of significance tests performed.

The EXACON analysis was conducted using the statistical package SLEIPNER (Version 2.1; Bergman et al. 2002). Confidence intervals for Cramér's *V* were calculated using an Excel calculator for non‐parametric effect sizes developed by Pautz et al. (2018). All other statistical analyses were conducted using IBM SPSS Statistics (Version 29).

### 2.2 Results

Analyses using Cochran's *Q* revealed group‐level changes in identity achievement, moratorium, and identity diffusion, but not in foreclosure, through emerging and established adulthood (see Table 2; full results for McNemar analyses are available at OSF). Post hoc comparisons showed that more individuals were in identity achievement at age 39 than at 25 or 29, fewer were in moratorium at age 39 than at 25, and fewer were in identity diffusion at age 39 than at 25 or 29. Statistically significant gender differences were found in identity status at age 25, *ꭓ* ^2^ (3, *N* = 105) = 17.9, *p* < 0.001, Cramér's *V* = 0.41, 95% CI [0.21, 0.67], with more women than men coded as identity achieved (ADJR = 3.6) and more men than women coded as identity diffused (ADJR = 3.1). Significant gender differences were also found at age 39, *ꭓ* ^2^ (2, *N* = 105) = 9.95, *p* = 0.007, *V* = 0.31, 95% CI [0.14, 0.56] (2 cells [33.3%] had expected counts less than 5, the minimum expected count was 1.40.); more women than men were coded as identity achieved (ADJR = 3.2) and more men than women were coded as foreclosed (ADJR = 3.0). No significant differences were found at age 29, *ꭓ* ^2^ (3, *N* = 105) = 10.94, *p* = 0.012, Cramér's *V* = 0.32, 95% CI [0.17, 0.58], or at age 33, *ꭓ* ^2^ (3, *N* = 105) = 6.03, *p* = 0.11, Cramér's *V* = 0.24, 95% CI [0.17, 0.49].

**TABLE 2:** Group‐level development in identity status across ages 25, 29, 33, and 39—descriptive data, Cochran's *Q* test, and McNemar test of significance of change.

Identity status	Age 25, *n* (%)	From age 25–29			Age 29, *n* (%)	From age 29–33			Age 33, *n* (%)	From age 33–39			Age 39, *n* (%)		McNemar, *ꭓ* ^2^					
		Stable, *n*	Change out *n*	Change in *n*		Stable, *n*	Change out, *n*	Change in *n*		Stable *n*	Change out *n*	Change in *n*		Cochran's *Q*	25–29	29–33	33–39	25–33	25–39	29–39
Identity achieved																				
Total	45 (42.9)	28	17	19	47 (44.8)	38	9	22	60 (57.1)	52	8	12	64 (61.0)	16.29***	—	—	—	—	8.757**	6.919**
Women	33 (58.9)	21	12	10	31 (55.4)	26	5	12	38 (67.9)	34	4	8	42 (75.0)							
Men	12 (24.5)	7	5	9	16 (32.7)	12	4	10	22 (44.9)	18	4	4	22 (44.9)							
Foreclosure																				
Total	34 (32.4)	22	12	19	41 (39.0)	24	17	12	36 (34.3)	29	7	9	38 (36.2)	1.97	—	—	—	—	—	—
Women	14 (25.0)	10	4	10	20 (35.7)	10	10	5	15 (26.8)	10	5	3	13 (23.2)							
Men	20 (40.8)	12	8	9	21 (42.9)	14	7	7	21 (42.9)	19	2	6	25 (51.0)							
Moratorium																				
Total	15 (14.3)	1	14	6	7 (6.7)	1	6	3	4 (3.8)	1	3	2	3 (2.9)	13.83**	—	—	—	—	*p* = 0.008^a^	—
Women	8 (14.3)	—	8	4	4 (7.1)	—	3	1	1 (1.8)	—	1	1	1 (1.8)							
Men	7 (14.3)	1	6	2	3 (6.1)	1	2	2	3 (6.1)	1	2	1	2 (4.1)							
Identity diffusion																				
Total	11 (10.5)	4	7	6	10 (9.5)	2	8	3	5 (4.8)	—	5	—	—	15.40**	—	—	—	—	*p* < 0.001^a^	*p* = 0.002^a^
Women	1 (1.8)	—	1	1	1 (1.8)	—	1	2	2 (3.6)	—	2	—	—							
Men	10 (20.4)	4	6	5	9 (18.4)	2	7	1	3 (6.1)	—	3	—	—							

*Note:* *N* = 105 (56 women and 49 men).

^a^ Exact sig. (two‐tailed), binominal distribution used.

** *p* < 0.01.

*** *p* < 0.001.

There were 39 different patterns of individual identity status change and stability across emerging and established adulthood (see Material S3 at OSF). In line with previous studies (e.g., Fadjukoff et al. 2016; Kroger et al. 2010) and theoretical propositions made by Waterman (1999) that optimal development from adolescence to adulthood includes exploration leading to commitments, we categorized changes in identity status as either progressive (D → F, D → M, D → A, F → M, F → A, and M → A) or regressive (F → D, M → D, A → D, M → F, A → F, and A → M). In this study, the largest group (35.2%, *n =* 37) followed stable patterns; that is, they were categorized into the same identity status at all four waves. The stable patterns consisted only of individuals coded as identity achieved or foreclosure, that is, statuses defined by established identity commitments. The second largest group (32.3%, *n* = 34) followed mixed patterns, meaning that they had made both progressive and regressive identity status transitions across the study's four waves. However, 18 of the individuals in the mixed group (17.1% of the total sample) showed a progressive trend over the last three waves. Thus, if considered together with the 24.8% (*n* = 26) who followed a progressive pattern over all four waves, progressive identity status patterns and patterns with a progressive trend together made up the largest group (41.9%, *n* = 44). A small number of individuals followed regressive patterns over all four waves of the study (7.6%, *n* = 8) or mixed patterns with a regressive trend over the last three waves (2.8%, *n* = 3).

**FIGURE 1:**
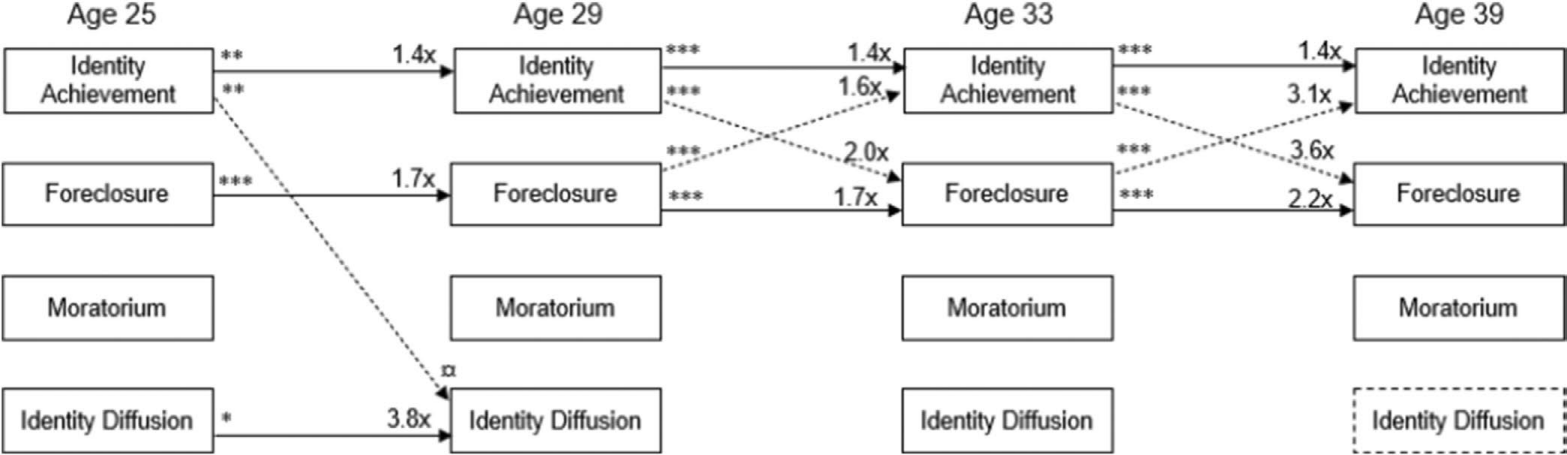
Typical and atypical patterns of individual stability and change in identity status between adjacent waves. *N* = 105. Solid and dotted arrows indicate typical and atypical patterns of individual identity status development, respectively. Numbers on the arrows indicate how many more or fewer times the pattern was observed than would be expected from chance alone. A “¤” indicates that no participants moved in the direction of the arrow. A dotted box indicates that no participants were categorized into that identity status. **p < 0.01, ***p < 0.001.

Person‐centered analyses of typical and atypical patterns of individual stability and change in identity status between adjacent waves of the study showed that stability in either identity achievement or foreclosure was more common than expected by chance between ages 25 and 29, 29 and 33, and 33 and 39 (see Figure 1; full results for EXACON analyses are available at OSF). Moreover, it was less common than expected by chance to change from identity achievement to identity diffusion between ages 25 and 29. It was also less common than expected by chance to change between foreclosure and achievement between ages 29 and 33 as well as 33 and 39. Because participants categorized into the same identity status across all four waves of the study were subject to further analysis (see Section 4), these patterns were also analyzed for gender differences; however, no statistically significant differences were found between participants who were stable in foreclosure (*n* = 15; 7 women, 8 men), stable in identity achievement (*n* = 22; 16 women, 6 men), and not stable in identity status, *ꭓ* ^2^ (2, *N* = 105) = 4.22, *p* = 0.12, Cramér's *V* = 0.20, 95% CI [0.14, 0.40].

### 2.3 Discussion

Findings from the quantitative part of this explanatory mixed‐methods study showed that stability in identity achievement or foreclosure across ages 25, 29, 33, and 39 was the most common type of developmental trajectory and that remaining in either of these statuses between adjacent waves of the study was more common than expected by chance. In addition to these stable patterns, progressive change was also evident as we saw a statistically significant increase in identity achievement and decreases in moratorium and identity diffusion from emerging through established adulthood. These findings generally align with previous research that indicates that the uncommitted statuses (i.e., identity diffusion and moratorium) tend to decrease over time while the committed statuses (i.e., foreclosure and achievement) tend to be stable or increase (Branje et al. 2021; Kroger et al. 2010; Meeus 2011). The results do not support the notion of cyclical movements between statuses as a common path toward identity development from emerging through established adulthood, such as moratorium‐achievement‐moratorium‐achievement (MAMA; Stephen et al. 1992) or foreclosure‐achievement‐foreclosure‐achievement (FAFA; Pulkkinen and Kokko 2000) cycles, although we cannot rule out the possibility that the participants had moved through such cycles between interviews.

The results from the quantitative part of this study suggest that identity development across emerging and established adulthood is characterized by establishing, or maintaining, identity commitments in central life domains. This pattern makes sense in light of what the transition from emerging to established adulthood entails; being a romantic partner, a parent, and/or employee might make it hard to engage in extensive exploration or remain diffused, thus forcing the individual to define central aspects of their identity. The high degree of individual stability in committed identity statuses suggests that the way in which commitments were formed (with or without previous exploration) had not changed, which is in line with the conceptualization of identity status as indicative of identity structure rather than more dynamic aspects of development (Kroger 2003). Thus, taken together, findings from this study support the idea of established adulthood as a time of identity solidification (Reifman and Niehuis 2022). Given the many identity‐relevant changes and challenges associated with established adulthood, these results raise questions as to how a stable identity is maintained and revised after commitments have been made. In the qualitative part of this study, we examined such processes of continued development for individuals who are stable in identity foreclosure and achievement.

#### 2.3.1 Limitations

The global, categorical identity status assessments used in this study are based on an extensive amount of interview data and provide insight into an individual's underlying identity structure at one moment in time, as well as the processes through which this structure developed. However, these assessments only provide a circumscribed picture of individual development as they, for example, lack information about domain‐specific patterns or the ongoing dynamics of different identity processes within an individual. The case‐based qualitative study part of this paper is an effort to overcome some of these shortcomings and provide a more nuanced and contextualized understanding of individual development. The data used in the current study is drawn from a longitudinal research project spanning almost 40 years. Although participant retention has been high in the project (between 94% and 81% during the last four waves), the small size of the sample can make true effects harder to detect as well as inflating small or even spurious associations (Button et al. 2013). In order to mitigate these risks, we have used a restricted alpha level of *p* < 0.01 and report effect sizes for all tests performed. Moreover, the results we obtained generally align with previous research findings (see Branje et al. 2021; Kroger et al. 2010; Meeus 2011), which strengthens the credibility of the results.

## 3 Qualitative Study

The research question examined in the qualitative part of this study was: What processes of continued identity development from ages 33 to 39 can be identified among individuals who are stable in committed identity statuses (foreclosure or identity achievement) across ages 25, 29, 33, and 39?

### 3.1 Method

#### 3.1.1 Participants

Participants included a subsample of individuals who had established commitments at age 25 and were categorized into the same identity status across ages 25, 29, 33, and 39 (*N* = 37), which involved participants who were stable in identity achievement (*n* = 22; 16 women, 6 men) and in foreclosure (*n* = 15; 7 women, 8 men).

#### 3.1.2 Data Analysis

To understand how established adults continue to develop their identity within stable identity status patterns, we conducted a longitudinal qualitative analysis of the participants' Identity Status Interviews from ages 33 and 39. In this analysis, we combined a case study approach (Willig 2013) with reflexive thematic analysis (Braun and Clarke 2022). The case study approach allowed for a holistic and contextualized understanding of individual development over time, while the use of reflexive thematic analysis enabled us to conceptualize meaningful patterns of development across cases. The analysis was conducted from a critical realist position (Pilgrim 2014) and we approached the data with an experiential orientation (Braun and Clarke 2022). As a starting point for the analysis, we used two conceptual models developed in previous studies from the longitudinal project that this study is part of. These models described processes of identity development for individuals who were stable in identity achievement or foreclosure from ages 25 to 29 (Carlsson et al. 2015) and 29 to 33 (Eriksson et al. 2020) in relation to three broad dimensions of development: identity commitments, identity narrative, and approach to social context. Each process was conceptualized as a continuum with a weakening and a deepening endpoint. In the present study, these dimensions, and the idea of conceptualizing processes of development on a continuum, were used as orienting concepts (Layder 1998). The key steps of the analysis are described below. For an extended methodology description, see Material S5 at OSF.

Case summaries were created for each of the participants focusing on similarities and differences between their interviews at age 33 and 39. In the first phase of the analytic process, a subset of these case summaries as well as interview transcripts were analyzed using reflexive thematic analysis (Braun and Clarke 2022). Given the extensive amount of data, comprising two interviews for each of the 37 participants, cases were added to the analysis in sets of four (one woman stable in A, one man stable in A, one woman stable in F, and one man stable in F) until no new material relevant to the research question was found in the data, resulting in a total of 12 cases. Participant cases were coded inductively by all authors for how their commitments, identity narrative, and approach to social context had developed from age 33 to 39, while also being open to processes that did not fit into the three‐dimensional model derived from previous papers (Carlsson et al. 2015; Eriksson et al. 2020). Codes were then clustered into a preliminary thematic model consisting of three processes of continued identity development, each conceptualized as a continuum with a weakening and a deepening endpoint. Based on this model, we constructed a codebook where, to facilitate coding, each process was divided into three scale steps representing a deepening, middle, and weakening development, respectively. Through an iterative process, the codebook was repeatedly evaluated against the 12 participant cases, resulting in further refinement in the definition of each process and their respective scale steps. The final version of the codebook is available as Material S4 at OSF.

In the second phase of the analytic process, all participant cases were coded in accordance with the codebook on the rating scale from deepening to weakening endpoints. This phase allowed us to quantify the development of participants in identity achievement and foreclosure and to assess the reliability of the coding. Moreover, it allowed us to substantiate the final presentation of each process in this paper with examples drawn from the full set of participant cases. First, all of the cases were rated by the first author. Thereafter, half of the total number of cases (*n* = 19) were randomly selected and rated by the third author, and inter‐rater reliability was measured through intraclass correlations (ICC) based on a mean‐rating (*k* = 2), absolute‐agreement, two‐way random‐effects model. These analyses indicated poor agreement for the processes *anchoring commitments in views of the self* (ICC = 0.18, 95% CI = −1.29–0.69) and *connecting oneself to older and younger generations* (ICC = 0.13, 95% CI = −1.25–0.66), and poor to moderate agreement for the process *story integration* (ICC = 0.59, 95% CI = − 0.07–0.84; Koo and Li 2016). Upon discussing and resolving all coding disagreements, we found that we sometimes arrived at different overall assessments when weighing the participants' domain‐specific development together; however, we had congruent impressions of their development in each domain and disagreements were always between adjacent steps on the continuum (except for one participant). We therefore concluded that the low inter‐rater reliability did not stem from conceptual issues but rather reflected the complexity of the coding procedure and possibly statistical issues related to lack of variability in the coding and a small sample size (Koo and Li 2016). Given these issues, we decided to establish reliability by means of consensus coding (Syed and Nelson 2015) the entire set of cases. The remaining 18 cases were therefore also rated by a second research team member (second author, 12 cases; third author, 6 cases), and disagreements were resolved through discussion between the two coders or the entire research team if needed.

### 3.2 Results

**FIGURE 2:**
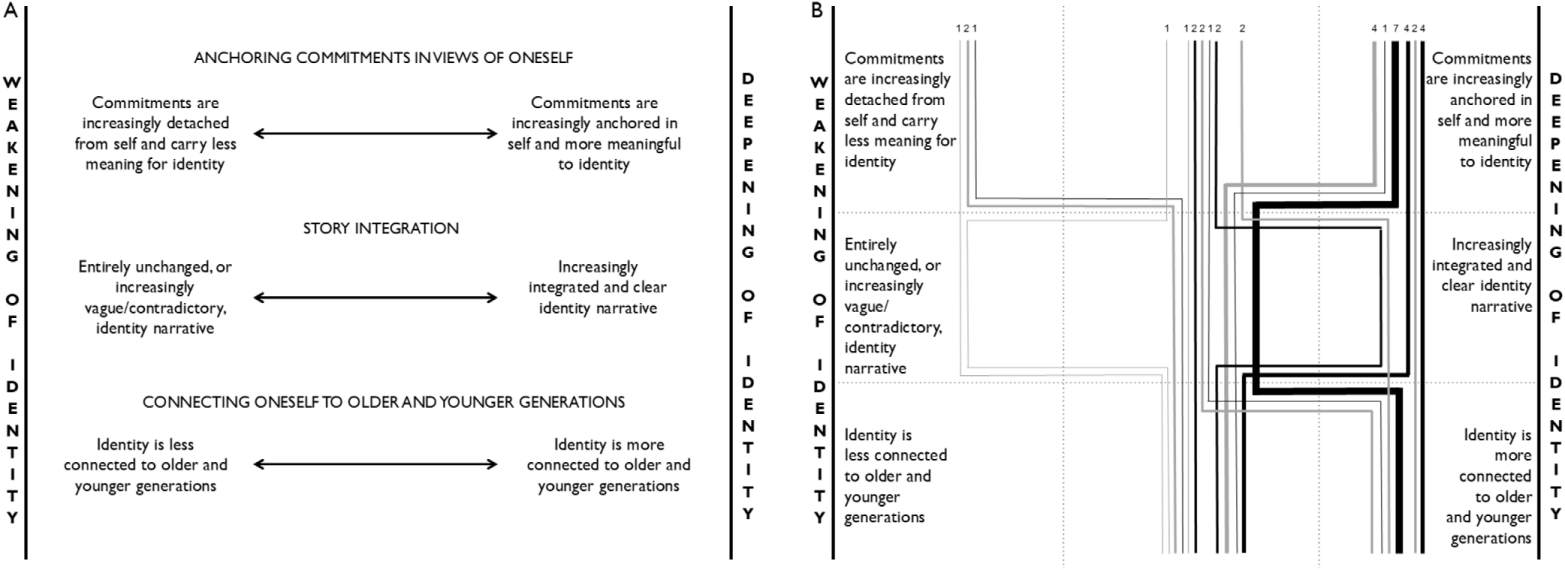
Processes of continued identity development for individuals who are stable in identity achievement or foreclosure from emerging through established adulthood. (A) Model of processes of continued identity development. (B) Individual patterns of development between ages 33 and 39 in relation to the three processes in the model. *N* = 37 (22 identity achievement, 15 foreclosure). The black bars represent participants assigned to identity achievement at all ages (25, 29, 33, and 39), and the gray bars represent participants assigned to foreclosure at all ages. The numbers on top of the bars indicate the number of participants who followed a specific developmental pattern.

The qualitative analysis of processes of continued identity development between ages 33 and 39 for individuals who are stable in identity foreclosure or achievement resulted in a model with three themes: (1) *anchoring commitments in views of oneself*, (2) *story integration*, and (3) *connecting oneself to older and younger generations* (see Figure 2A). Each theme described a process of identity development and was conceptualized as a continuum with a weakening and a deepening endpoint. The deepening endpoint in all three processes represented increased engagement in that process between the interviews and an identity that had evolved toward becoming richer and more integrated, yet flexible. Conversely, the weakening endpoint represented decreased engagement in that process and an identity that had become more rigid, vague, or closed off. For Processes 1 and 3, the midpoint of the continuum represented similar engagement in that process across the interviews, thus signaling developmental stability. For the second process, *story integration*, a small change in the narrative was needed in order to be rated at the midpoint, based on the theoretical proposition that people need to continuously revise their stories about themselves in light of new experiences in order to maintain stability (McAdams 2013). The three processes in the model are presented below along with illustrative excerpts from participants.

#### 3.2.1 Process 1: Anchoring Commitments in Views of Oneself

This process concerns development in how the individual anchored their identity commitments in their overall views of themselves. It also involves development in the meaning they ascribed to their commitments for their identity and life. Given that the participants had been identity foreclosed or achieved since age 25, most still upheld identity commitments at age 39 that were similar to those they had established earlier in life. The process of *anchoring commitments in views of oneself* does therefore not focus mainly on commitment change but rather on how the individual managed to maintain their commitments as personally relevant and meaningful aspects of their identity.

For participants rated at the deepening end of this process, their commitments appeared to have become more integral to how they viewed themselves and chose to live their lives. This development was expressed through reflecting more and in greater depth on how their commitments aligned with other aspects of themself, such as self‐prescribed personality traits, interests, skills, and values; for example, describing their commitments concerning romantic relationships as “It's probably just my personality, to have this continuity and security, this stable foundation in some way that suits me.” One of the participants who showed a deepening development in this process was Markus. When asked at age 33 why he had chosen his profession, he answered:

I didn't. The whole point of choosing to study science and then industrial economics is that it's the broadest profile you can find [….] and the management company I started working at had an offer in real estate and then I was recruited by [company]. So, it's all a big coincidence.

At age 39 he was working within the same industry but had started his own company. Although he still maintained that his career path was largely coincidental, he now described his profession more in terms of how it aligned with his views of himself:

I enjoy it partly because my interests and abilities are a good fit for it. [….] you get to know yourself over time. I'm pretty good at spatial things and turning cubes and reading maps and so on. And I quite like numbers and structure and stuff like that. And that fits in very well when you make a floor plan in a building.

Like Markus, most participants with a deepening development seemed to draw more confidence, security, and meaning from their commitments at the second interview, which came across in a tendency to describe their commitments in greater detail than before and to be more certain about their ability to live in accordance with them. However, for some participants, a deepening development could also involve working more actively than before toward solving conflicts between their commitments, their views of themselves, and their lives. Moreover, although most participants with a deepening development had similar commitments at ages 33 and 39, a few described adjusting or changing their commitments entirely since their last interview (yet were categorized into the same identity status as they expressed similar levels of exploration and commitment at both interviews). These participants reflected on their commitment change in relation to how they viewed themselves as a person, and changing their commitments appeared to be motivated by a wish to establish commitments that were more meaningful to their identities and lives.

For participants rated at the weakening end of the process *anchoring commitments in views of oneself*, commitments appeared to be increasingly detached from how they viewed themselves and lived their lives, and to carry less meaning for their identities. A weakening development could be expressed in the narratives by reflecting less or in a shallower way on their commitments in relation to how they viewed themselves as a person, instead depicting their commitments as dictated by cultural norms, a mere adaptation to external circumstances, or a rigid set of rules. Moreover, it could involve an inability to update their commitments or how they were translated into action in a way that aligned with the participant's current life situation, as shown in the case of the participant Kalle. At both ages 33 and 39, he described parenthood as central to his identity and was committed to being a dedicated father. However, at age 39 he seemed to struggle with no longer being able to help his children with schoolwork, an activity he described as important to his parental identity in both interviews:

What I feel I would like to be more involved in is [their] schoolwork. Well, my daughter finishes her homework before we get home, she's a real schoolgirl. [….] And I guess the son prefers to do it himself. [….] So, it's not easy.

As illustrated by the case of Kalle, a weakening development in this process often involved participants speaking of their commitments with less conviction and confidence than before.

As shown in Figure 2B, most participants stable in identity achievement showed a deepening development in the process *anchoring commitments in views of oneself* between interviews (*n* = 16, 73%), and only one was rated at the weakening endpoint. The foreclosed participants showed greater variability, with six individuals (40%) exhibiting signs of a deepening development and three (20%) a weakening development.

#### 3.2.2 Process 2: Story Integration

The process of *story integration* focuses on changes in how the individual made sense of and communicated who they were through the story they told about themselves. Specifically, this process concerns the participant's development in their ability to integrate different and sometimes contradictory elements of their interview narratives—such as accounts of experiences, views, and life choices—into a clear story of who they were. Importantly, the process of *story integration* does not focus on changes in the length or breadth of the participants' interview narratives; rather, it focuses on changes in the identity relevance of the content they chose to include and how they made sense of that content.

The deepening end of this process represented cases with stories that had become increasingly woven together and clear accounts of who the participant was. This could involve being more effective in communicating main themes of the story, for example by omitting information that was less central to their identity or by integrating elements from different identity domains to emphasize main themes. Greater integration and clarity could also entail clarifying how one's views and life choices come to be, for example by elaborating on underlying motivations or the influence of different life events. An example of a deepening development in this process was found in the case of the participant Niklas. At age 33, he described pursuing career options in different fields of work and ending up in an administrative position in health care mainly due to coincidence. At age 39, now in a management position at the same company, he reflected on how his chosen career, although initially coincidental, also made sense in light of his history:

I've probably always been kind of into this caregiving thing from my upbringing and from school before and I've also liked to coach or just teach others, so to speak. Which I now get an outlet in as a manager instead. So, like, educating or helping others has always been a part of my life in one way or another.

The theme of being a person who likes to help others grow ran through Niklas's narrative about his professional development at age 39 and was also extended to his family life, as he described himself as a parent committed to helping his children develop into their best versions of themselves. As in the case of Niklas, a deepening development in this process often resulted in a story that came across as more processed than before, as if the participant had gained enough perspective on themselves and their life to be able to make connections between and draw conclusions about their experiences. For some, a deepening development could also entail telling a story that was richer and more complex, as it contained more elements and nuances.

The weakening end of the process of *story integration* represents cases with stories that came across as increasingly vague or contradictory accounts of the individual's identity. For participants rated at the weakening endpoint, this development involved adding new elements to their interview narrative that were not integrated into their overall story or by not elaborating on major life events that had taken place between interviews, thus making it hard to grasp the personal meaning of their experiences, views, and life choices for their identities. A weakening development could also mean providing a sparser story, for example by omitting parts of one's interview narrative that had seemed central to their identity at age 33 without adding any new identity‐relevant elements. Louise was one of the participants who showed signs of weakening *story integration*. At age 33 she elaborated on her views on romantic relationships and how they had developed, for example describing an earlier period in life when she had wanted to be single to know that she could manage on her own. At age 39, she had omitted this narrative about her time as single and simply stated “I think I've always known [that I want to be in a relationship]” while not adding any new elements to her story. This development was visible in other parts of her interview narrative as well, making it come across as a less clear account of her identity.

For the second process, Figure 2B shows that nearly half of the identity achieved participants (*n* = 10, 45.5%) showed increased *story integration* between the interviews and none showed a weakening development. Among the foreclosed participants, a few were rated at the deepening (*n* = 4, 26.7%) and weakening (*n* = 2, 13.3%) endpoints while the majority did not show substantial change in their level of *story integration*.

#### 3.2.3 Process 3: Connecting Oneself to Older and Younger Generations

The process of *connecting oneself to older and younger generations* concerns changes in how the individual understood, defined, and actualized their identity in relation to others. Reflections involving older generations often included the participant's parents; however, grandparents, great grandparents, and other older relatives and family members were also mentioned. Reflections regarding younger generations mainly revolved around the participant's children, but could also include other children in their social context or, more broadly, future generations to come.

The deepening end of this process represents cases that had developed toward connecting themselves and their identities more explicitly in relation to older and younger generations. This development involved reflecting more or in greater depth on how their views and life choices were influenced by and affected others. To be rated at the deepening end of this process, increases in generational reflections between ages 33 and 39 also had to carry identity‐relevant implications for the participant, for example to deepen self‐understanding, inform self‐definition, or guide behavior and life choices in some way. Max was one of the participants who connected his identity more to older and younger generations at age 39 compared to age 33. At age 33, he and his partner were expecting their first child and he stated that he wanted to be a less authoritarian parent than his own father had been. At age 39, he had deepened his reflections around this theme and described how becoming a parent had motivated him to actively develop his parenting identity and practices:

I don't want to be an authoritarian parent. I had a father who was very authoritarian when I was growing up, so I've read a lot of books about parenting and so on and tried to find out “how it should be”. [….] So, I've tried to work against myself there, that I know that I'll… I probably have this authoritarian side to me, and I don't want to color my children with that.

As exemplified by Max's case, connecting oneself more to older and younger generations often involved making more connections between older and younger generations as well. This could mean reflecting on what to pass on or not from one's parents to one's children or becoming aware of similarities with one's parents through interactions with one's children.

The weakening end of the process of *connecting oneself to older and younger generations* would theoretically represent cases with a development toward reflecting less on themselves and their identities in relation to others, or who described their identities more explicitly in isolation from older and/or younger generations than before. However, no participants showed enough of a weakening development to be rated at this endpoint.

As illustrated in Figure 2B, just over half of the identity achieved participants (*n* = 12, 54.5%) showed a deepening development in the third process, whereas this development was slightly less common in the foreclosed group (*n* = 6, 40%).

#### 3.2.4 Individual Patterns of Development Across the Three Processes

The participants exhibited 11 different developmental patterns in relation to the three processes (see Figure 2B). Of the identity achieved participants, all but one were rated at the deepening or middle part of the model on all three processes, with the majority (*n* = 15, 68.2%) being rated at the deepening endpoint in at least two of the processes. Individuals rated at foreclosure showed more varied patterns of development; however, most (*n* = 10, 66.7%) were rated at the middle point in at least two of the three processes.

### 3.3 Discussion

In the qualitative part of this study, we examined processes of continued identity development from age 33 to 39 among individuals who are stable in identity achievement or foreclosure from emerging through established adulthood. The analysis resulted in a model with three themes, each describing an identity process: *anchoring commitments in views of oneself*, *story integration*, and *connecting oneself to older and younger generations*.

The process of *anchoring commitments in views of oneself* describes development in the individual's approach toward integrating their identity commitments with their overall views of themselves and in the meaning they ascribe to their commitments for their identity and life. Previous studies from the longitudinal project that this study is part of found development in how individuals adjusted their commitments in response to external changes (ages 25–29; Carlsson et al. 2015) and internal needs (ages 29–33; Eriksson et al. 2020). Interestingly, changing one's commitments seemed less central to development between ages 33 and 39 than earlier; instead, we saw development mainly in how commitments were maintained as a meaningful part of the individual's identity. This process relates to previous findings in adolescent and emerging adult samples on the importance of commitment quality for adjustment and well‐being; for example, making commitments that feel meaningful (Soenens et al. 2011), identifying with one's commitments (Luyckx et al. 2005, 2006), and viewing them as personally expressive (Waterman 2004, 2007). The present study thus shows how individuals in established adulthood through the process of *anchoring commitments in views of oneself* maintained, and deepened, the quality of their commitments although the commitments (or the individual's life circumstances) had not really changed. The shift from changing one's commitment to instead maintaining them might reflect the increased stability and decreased opportunities for exploration that are characteristic of established adulthood (e.g., Mehta et al. 2020), and possibly greater self‐knowledge and confidence due to further immersion in important social roles (Mehta and LaRiviere 2022). For those who actually had changed their commitments, reflecting on this change in relation to their overall views of themselves could be seen as a way of integrating their new commitments into their identity. The process *anchoring commitments in views of oneself* thus seemed to function both as an identity maintenance and re‐establishment process.

The second process in the model, *story integration*, focuses on a narrative dimension of identity development and describes changes in how the individual makes sense of and communicates who they are through the story they tell about themself. This process captured development in how different narrative elements (e.g., accounts of experiences, views, and life choices) were logically connected and related to an overarching theme, which relates to the narrative identity concept thematic coherence (Habermas and de Silveira 2008; Reese et al. 2011). It also involved development in how individuals interpreted their experiences and related them to their identity, which is in line with the concept of autobiographical reasoning (Habermas and Bluck 2000). In previous studies from the longitudinal project that this study is part of, development in this narrative dimension of the model involved adding new elements of meaning (ages 25–29; Carlsson et al. 2015) and temporal and thematic integration (ages 29–33; Eriksson et al. 2020). Similar to findings by Eriksson et al. (2020), we also found development in *story integration*. However, how this integration was achieved had somewhat shifted: Between the ages of 29 and 33 in Eriksson et al. (2020) many had added new details to their story, whereas in this study between ages 33 and 39 many highlighted identity‐relevant themes both by adding information and omitting less relevant parts of their narrative thus making the story more coherent. The development seen in this narrative dimension of the model across emerging and established adulthood could thus be described as a gradual shift from expanding the story in one's late 20s to refining it in one's late 30s. This finding aligns with the literature on narrative identity development: It is during emerging adulthood that people start constructing a narrative identity, which then becomes increasingly complex and coherent throughout established adulthood and midlife as autobiographical reasoning (McAdams and Olson 2010) as well as thematic coherence (Peters et al. 2025; Reese et al. 2011) increase. Moreover, the shift from expansion to refinement may also reflect the fact that many formative life events tend to take place in people's 20s to early 30s (e.g., leaving the parental home, committing to a first serious relationship, entering the job market, starting a family), events that are often personally meaningful as well as culturally expected chapters in people's life stories (Rubin and Berntsen 2003). The present study contributes to this literature by showing that adding new elements to one's narrative identity may be a less prominent aspect of development in established adulthood than further integrating the elements that are already there, and that this integration can function as an identity maintenance process (Mitchell et al. 2021).

The third process in the model, *connecting oneself to older and younger generations*, concerns change in how the individual understands, defines, and actualizes their identity in relation to older and younger generations. This process highlights the psychosocial nature of identity development emphasized by Erikson (1963, 1968), and the focus on younger generations is in line with his description of generativity as the main developmental task during this part of the lifespan. Interestingly, development in this dimension of the model, which focuses on the individual's approach to others and society, was for many tied to more proximal social contexts between ages 33 and 39 than in previous ages. Between ages 25 and 29, the aspects concerning approach to social context revolved around how participants related to social norms and expectations when they developed their personal life direction (Carlsson et al. 2015), and between ages 29 and 33 around how they took part in society beyond their immediate social context (Eriksson et al. 2020). The increased focus between ages 33 and 39 on more proximal social contexts could be understood in light of the high dual demands in work and family life that are characteristic of established adulthood, which might require individuals to focus on main social roles and responsibilities during this *rush hour of life* (Knecht and Freund 2016; Mehta et al. 2020). This study shows how these important social roles may spur reflection and ongoing identity work, and how established adults can maintain, deepen, and re‐establish their identity by connecting their identity to important others during this phase of life.

The extent to which individuals engage in these three processes of continued development varied in the present study. For individuals who are stable in identity achievement, we found only one instance of a participant being rated at the weakening endpoint in any of the processes, with the majority being rated at the deepening endpoint in at least two of the processes. While individuals coded as foreclosed showed more varied patterns of development, most (*n* = 10, 66.7%) were rated at the middle point in at least two of the three processes. Although no statistical analyses of group differences could be performed, these findings suggest that establishing commitments in emerging adulthood through exploration may be a facilitating—but not crucial—factor for the continued deepening of identity in established adulthood. It would be valuable for future research to further investigate these potential differences between the achievement and foreclosure statuses in larger samples, and to explore their potential links to other aspects of psychosocial functioning.

#### 3.3.1 Limitations

Although the codebook approach to qualitative analysis taken in this study enabled us to compare the development of participants in identity achievement and foreclosure, we acknowledge that this approach may limit the level of nuance and detail that we were able to describe and conceptualize in our analysis. Moreover, the coding of the participants' development in relation to the three processes in our model was arrived at by weighting together their development in each identity domain (work, romantic relationships, parenthood, and work‐family priorities) following a set of guidelines (see Material S4 at OSF). The global approach allowed for a more holistic and conceptually clear model than if we had focused on the participants' domain‐specific development. However, many participants did show somewhat mixed patterns of development in each process across the four identity domains included in the interview, and this variability was not captured by our approach. Future studies could therefore complement the findings from this study by examining identity processes in established adulthood in relation to specific identity domains or social roles, or identity processes in uncommitted statuses (see e.g., Carlsson et al. 2016; Larsson et al. 2020), and by conducting in‐depth qualitative work from a Big Q paradigmatic position. This would help to further elucidate variation within and between individuals, which would be a valuable contribution to the emerging research field of identity development in established adulthood.

In the present study, participant cases were coded inductively for how they had developed from age 33 to 39 in relation to three dimensions identified in previous studies from the same longitudinal project (i.e., commitments, identity narrative, and approach to social context; Carlsson et al. 2015; Eriksson et al. 2020), while also being open to other processes of development that did not relate to them. This approach made the analysis sensitive to the phase‐specific development of established adults and helped highlight prominent developmental features of this phase, which was in line with our aim. However, we do expect that the processes identified in this paper, as well as in Carlsson et al. (2015) and Eriksson et al. (2020), are present across adulthood, rather than represent discrete categories tied to specific age ranges. Examining the presence of the specific processes across emerging and established adulthood would be a valuable step in highlighting how these processes change continuously over time.

## 4 Conclusions

The findings from the quantitative part of this study show that at the group level, identity status change toward maturity takes place from emerging to established adulthood. However, making identity commitments already at age 25 and remaining in the same identity status across all waves of the study was the most common individual pattern of development. The qualitative analysis shows that processes of continued identity development also take place within these stable identity status patterns—that there is change within stability. These processes seem to help individuals maintain and deepen their identity, but in some cases also re‐establish their identity after having gone through more profound change. The double function of the processes identified in the present study suggests that the distinction between maintenance and re‐establishment processes put forth by Mitchell et al. (2021) is not always clear‐cut when one examines the lived experience of individuals from the normal population. It may be the case that this distinction is more meaningful in examinations of clinically impairing identity issues (e.g., in response to traumatic life events) than in the type of change processes observed in the current study, which are perhaps better described as processes of identity revision (cf. Kroger 2002) than re‐establishment.

Taken together, the findings from the current study thus highlight the next step in identity development for individuals who have long ago formed identity commitments in important life domains: They show the processes through which individuals may maintain and revise a stable identity, or fail to do so, in established adulthood. These processes can be conceptualized as development in how commitments are anchored in views of the self, in integration of the identity narrative, and in how individual identity is connected to younger and older generations. Together, the processes show that development at this time of life is not mainly centered around changing or adding new parts to one's identity but rather on further integrating the elements that are already there and making new meaning around them, in relation both to one's personal characteristics and history as well as to important others.

## Author Contributions

**Hanna Larsson:** conceptualization, formal analysis, investigation, methodology, project administration, visualization, writing – original draft, writing – review and editing. **Johanna Carlsson:** conceptualization, formal analysis, investigation, methodology, visualization, writing – review and edits. **Py Liv Eriksson:** conceptualization, formal analysis, funding aquisition, investigation, methodology, visualization, writing – review and edits. **Moin Syed:** conceptualization, writing – review and edits. **Maria Wängqvist:** conceptualization, formal analysis, investigation, methodology, visualization, writing – review and edits. **Ann Frisén:** conceptualization, formal analysis, funding aquisition, investigation, methodology, project administration, supervision, visualization, writing – review and edits.

## Funding

This study was supported by the Swedish Research Council for Health, Working Life and Welfare (FORTE) under grant numbers (2005‐0874, 2011‐00722, 2014‐00265, and 2019‐00446).

## Disclosure

The participants of this study did not give consent for their data to be shared publicly, so due to the sensitive nature of the research, supporting data is not available. This study was not pre‐registered.

## Ethics Statement

The Regional Ethical Review Board in Gothenburg approved all data collections: protocol numbers 311‐06 (Wave 8), 206‐11 (Wave 9), 263‐15 (Wave 10), and 2020‐01146 (Wave 11). The authors have no conflicts of interest to disclose.

## Data Availability

The participants of this study did not give consent for their data to be shared publicly, so due to the sensitive nature of the research, supporting data is not available.
